# Image-Based 3D Reconstruction in Laparoscopy: A Review Focusing on the Quantitative Evaluation by Applying the Reconstruction Error

**DOI:** 10.3390/jimaging10080180

**Published:** 2024-07-24

**Authors:** Birthe Göbel, Alexander Reiterer, Knut Möller

**Affiliations:** 1 Department of Sustainable Systems Engineering—INATECH, University of Freiburg, Emmy-Noether-Street 2, 79110 Freiburg im Breisgau, Germany; alexander.reiterer@mail.inatech.uni-freiburg.de; 2 KARL STORZ SE & Co. KG, Dr.-Karl-Storz-Street 34, 78532 Tuttlingen, Germany; 3 Fraunhofer Institute for Physical Measurement Techniques IPM, 79110 Freiburg im Breisgau, Germany; 4 Institute of Technical Medicine—ITeM, Furtwangen University (HFU), 78054 Villingen-Schwenningen, Germany; knut.moeller@hfu.eu; 5 Mechanical Engineering, University of Canterbury, Christchurch 8140, New Zealand

**Keywords:** 3D reconstruction, laparoscopy, quantitative evaluation, reconstruction error

## Abstract

Image-based 3D reconstruction enables laparoscopic applications as image-guided navigation and (autonomous) robot-assisted interventions, which require a high accuracy. The review’s purpose is to present the accuracy of different techniques to label the most promising. A systematic literature search with PubMed and google scholar from 2015 to 2023 was applied by following the framework of “Review articles: purpose, process, and structure”. Articles were considered when presenting a quantitative evaluation (root mean squared error and mean absolute error) of the reconstruction error (Euclidean distance between real and reconstructed surface). The search provides 995 articles, which were reduced to 48 articles after applying exclusion criteria. From these, a reconstruction error data set could be generated for the techniques of stereo vision, Shape-from-Motion, Simultaneous Localization and Mapping, deep-learning, and structured light. The reconstruction error varies from below one millimeter to higher than ten millimeters—with deep-learning and Simultaneous Localization and Mapping delivering the best results under intraoperative conditions. The high variance emerges from different experimental conditions. In conclusion, submillimeter accuracy is challenging, but promising image-based 3D reconstruction techniques could be identified. For future research, we recommend computing the reconstruction error for comparison purposes and use ex/in vivo organs as reference objects for realistic experiments.

## 1. Introduction

Laparoscopy enables minimally invasive surgery (MIS), which reduces patience’s healing duration and surgical trauma [1]. Mostly, it is used therapeutically for resectioning tumors and organs, e.g., parts of the liver or gall bladder, or to diagnose malicious tissue, e.g., peritoneal metastases [2] or endometriosis [3]. Future laparoscopic applications cover image-guided surgery (augmented reality (AR)) [4] or (autonomous) robot-assisted interventions [5]. Image-based 3D reconstruction, which requires accurate depth measurement (distance from camera to object) as well as camera localization, enables these applications by creating a virtual 3D model of the abdomen. A robotic laparoscopic system with 3D measurement ability is offered by Asensus Surgical US, Inc. (Durham, North Carolina, USA) [6], which is based on stereo camera reconstruction.

To provide a recommendation for future research regarding high accuracy 3D reconstruction techniques, literature from 2015 to 2023 was reviewed [7]. Only articles that measure the reconstruction error—especially the root mean squared error (RMSE) and the mean absolute error (MAE)—were considered. The purpose of this work is to give a statement about the image-based 3D reconstruction technique with the highest accuracy.

Section 2 explains the literature search and gives an overview of the quantitative evaluation. Section 3 presents the review’s results, which will be discussed in Section 4, while Section 5 concludes the paper. Before it is continued with Section 2, the image-based 3D reconstruction techniques and related work are demonstrated.

### 1.1. Image-Based 3D Reconstruction Techniques Used in Laparoscopy

**Figure 1:**
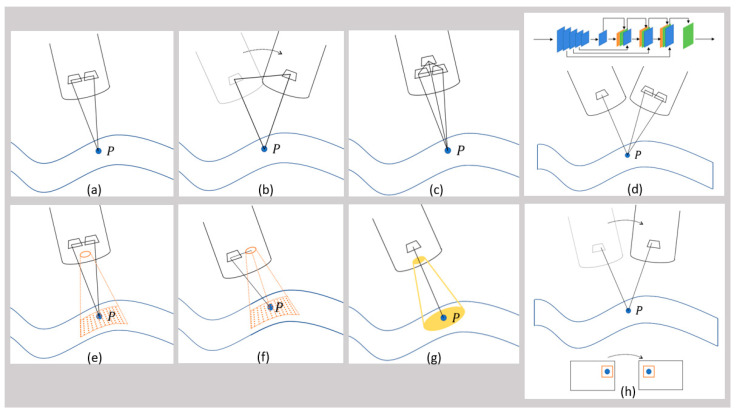
Schematic overview of different image-based 3D reconstruction techniques. (**a**) Stereo vision, the point P is captured by both cameras; (**b**) Structure-from-Motion (SfM), the point P is captured by the mono camera from two different perspectives; (**c**) trinocular vision, the point P is captured by the three cameras; (**d**) deep-learning-based approaches (DL), the point P is captured by either a mono or stereo camera, the blue, green and orange rectangles represent different layers of a DL network; (**e**) active stereo vision with structured light (SL stereo), the point P is captured by the stereo camera while a pattern (represented by the orange dots) is projected onto the surface; (**f**) structured light with monocular vision (SL mono), the point P is captured by the mono camera while a pattern (represented by the orange dots) is projected onto the surface; (**g**) Shape-from-Shading (SfS), the point P is captured by the mono camera and the white light is represented by the yellow circle; (**h**) Simultaneous Localization and Mapping (SLAM), the point P is captured by the mono camera from two different perspectives, the point P in the image is matched to the point P in the neighboring image.

Image-based 3D reconstruction techniques can be divided into passive (only image is used) and active (additionally, external light is brought onto the object’s surface) methods [8]. Most of them make use of triangulation to estimate depth (distance from camera to object). Shape-from-Motion (SfM) [9] (Figure 1b) and Shape-from-Shading (SfS) [10] (Figure 1g) belong to the category Shape-from-X (SfX) and are declared as passive methods. Stereo vision [4] (Figure 1a), trinocular [11] (Figure 1c) or multicamera systems [12], as well as Simultaneous Localization and Mapping (SLAM) [13] (Figure 1h) are passive methods as well. Deep-learning (DL) approaches (Figure 1d) are trained with either mono- or stereoscopic images and can also be assigned to passive methods [14,15,16,17,18,19,20,21,22,23,24]. Active methods are Time-of-Flight (ToF) cameras [25] and Structured Light (SL) [26] (Figure 1e (SL stereo), f (SL mono)). ToF will not be presented in this paper because within the last nine years, we could not find any paper using ToF for 3D reconstruction in laparoscopy. At last Smart Trocar^®^ and Light Field Technology must be mentioned. The first one is a trocar equipped with cameras to record the laparoscope’s and instruments’ positions [27]. Light Field Technology is another passive method but is barely used in laparoscopy [28].

The category SfX contains algorithms, which consider monocular images. The most common technique within this category is SfM. Here, the motion of the camera causes image pairs, which lead to triangulation. Through motion, the system can capture an object from different perspectives. The distance between two images (stereo base or base distance) is variable and depends on the motion [8]. The method SfS analyzes the pixel intensity of monocular images to estimate depth—the more intense, the closer the object [8,29].

Stereo vision describes a system with two cameras and a fixed stereo base. Stereo matching algorithms search for corresponding points in the image pair, which can be used to calculate the disparity and by applying triangulation, depth can be determined [8,30,31]. While stereo vision estimates depth, SLAM tracks the camera positions. It originates from the research area of mobile robots and requires feature detection and loop closing. Through camera movement, features change their pixel position on the image sensor, which correlate with the camera position change [8,32,33].

Trinocular systems belong to the category of multicamera systems and operate comparable to stereo cameras, but with additional cameras [11]. In contrast to this, ToF cameras (active technique) are based on emitting light impulses, which return after reflection from the object’s surface. The time from emitting to returning correlates with the object distance (depth) [8,25]. The technique SL requires a projector, which creates a pattern on the surface, which is captured by either a mono or stereo camera. Patterns could be dots, grids, lines, individual or random dot patterns. Instead of the white light image, the matching algorithms analyze the pattern and triangulation is calculated between two cameras (SL stereo), or between the mono camera and the projector (SL mono) [8,34].

AI approaches contain depth networks (neural networks), which are trained with datasets of either monocular or binocular images. Often, the models are based on self-supervised or unsupervised convolutional neural networks. SLAM algorithms could complement in parallel for localization [14,15,16,17,18,19,20,21,22,23,24,35,36,37,38,39].

### 1.2. Related Work

Several reviews of laparoscopic image-based 3D reconstruction could be found—without offering the reconstruction error [8,40,41,42,43] and with. The ones including the reconstruction error are presented in the following.

Ref. [44] generated a surface reconstruction of organ phantoms (liver, kidney, and stomach) by using a ToF camera and by applying stereo vision to compare it with CT data. Stereo vision outperformed the ToF camera with an accuracy of 1.1–4.1 mm (RMSE) vs. 3.7–8.7 mm (RMSE). SfM was part of the comparison as well, which resulted in 3.5–5.8 mm (RMSE). Ref. [45] presented SfM, SLAM, SfS, DSfM, binocular, trinocular, and multi-ocular systems, SL and ToF. They claim that SfM, refined by SfS, could deliver an accuracy of 0.1–0.4 mm (RMSE) on simulated objects, stereo vision could generate an accuracy of 0.45 mm (RMSE) when scanning a skull, a trinocular approach could reconstruct a piece of meat with an accuracy of −0.5 ± 0.57 mm (MAE), a multi-ocular approach resulted in near millimeter accuracy, and a SL approach could reconstruct the inside of an hollow object with <0.1 mm accuracy (RMSE), whereas a ToF endoscope could deliver a 4.5 mm accuracy (RMSE) when reconstructing a lung surface compared to 3.0 mm by stereo vision. Ref. [46] compared four stereo vision approaches using laparoscopic stereo video sequences.

However, it is unclear which approach delivers the highest accuracy because there are not enough data, and these references did not consider deep-learning approaches. Due to those gaps, this review collects more data and considers deep-learning approaches.

### 1.3. Research of Image-Based 3D Reconstruction in Laparoscopy since 2015

The data presented in this article are an extension of the IFAC conference paper [7]. Compared to Ref. [7], also data from 2023 are listed, and a classification of the collected data regarding reference object, ground truth acquisition, deep-learning networks, and stereo- or mono cameras are made.

Before going into details of the quantitative evaluation, the distribution of 3D reconstruction techniques since 2015 is demonstrated. Therefore, all 72 articles (with and without quantitative evaluation) have been sorted regarding 3D reconstruction technique and their publication dates (see Table 1, some articles are mentioned multiple times, which explains the difference between 76 and 72 articles) [7]. Stereo vision was very present in the years 2015–2018 and became less afterwards, whereas AI approaches became increasingly popular having the highest portion in 2021, 2022, and 2023. The distribution is as follows: Stereo vision 26.3%, AI 26.3%, SL 14.5%, SfM 13.2%, SLAM 13.2%, SfS 1.3%, Smart Trocar 1.3%, Trinocular 1.3%, Light Field Technology 1.3%, Multicamera 1.3%, and ToF 0%.

## 2. Methods

This chapter presents the search strategy and the metrics usable for the quantitative evaluation of the reconstruction error.

### 2.1. Search Strategy

**Figure 2:**
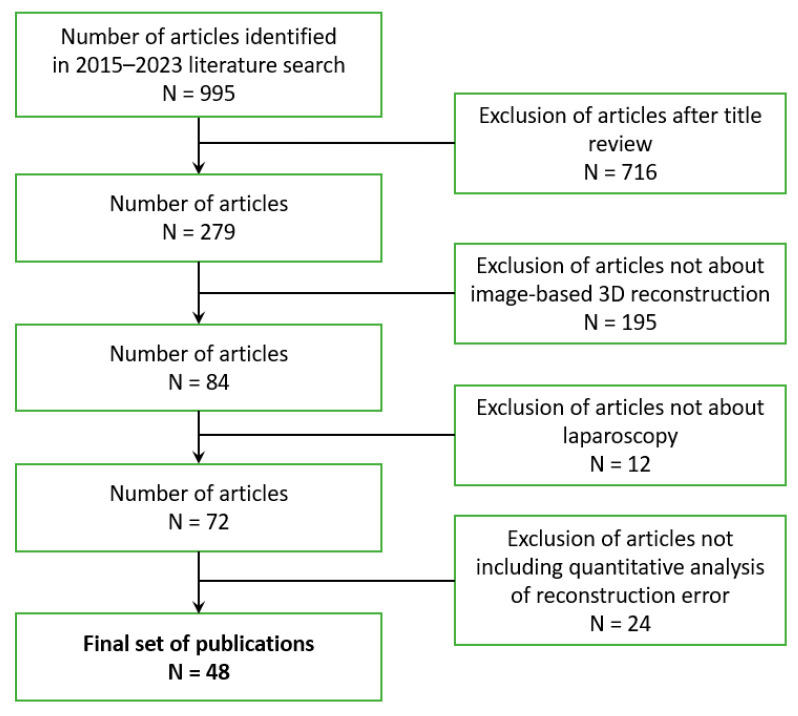
Paper exclusion tree.

For literature search, the engines “PubMed” and “google scholar” were applied by setting the keywords to “3D reconstruction laparoscopy”, “three-dimensional reconstruction laparoscopy”, and “surface reconstruction laparoscopy”, while the year of publication was set to 2015–2023. Articles were excluded if not about image-based 3D reconstruction techniques, if not about laparoscopy, and if a quantitative analysis is missing (see Figure 2) [7]. In total, 995 articles were found, which were filtered by reading the title, the abstract, and the keywords. Out of those, 716 articles were excluded after title review, 195 articles were excluded because they did not talk about image-based 3D reconstruction, and 12 articles were removed due to the missing topic of laparoscopy. Exclusion criteria led to 72 articles. The last criterion—the presence of the quantitative evaluation of the reconstruction error, especially the RMSE and the MAE—excluded 24 articles. Thus, 48 articles were considered in this review containing studies of image-based 3D reconstruction in laparoscopic applications from full-text articles, conference-proceedings, and abstracts.

For the review process of the literature, we followed the guidelines of Refs. [96,97] and considered the steps of topic formulation, study design, data collection, data analysis, and reporting. This includes the objectives of the review, the creation of a protocol, which describes procedures and methods to evaluate the published work, as well as a standardized template in Excel for the data analysis of the published quantitative evaluation criteria and values. Reporting requires the presentation, interpretation, and discussion of the results as well as the description of implications for future research. The data collection includes the following items: 3D reconstruction technique, reference object, acquisition of ground truth, number of cameras (mainly mono and stereo camera), method of camera localization, metric of quantitative evaluation, number of shots (single vs. multi), number of image sequences/frames/datasets, and image resolution.

### 2.2. Metrics for Quantitative Evaluation of 3D Reconstructions

**Figure 3:**
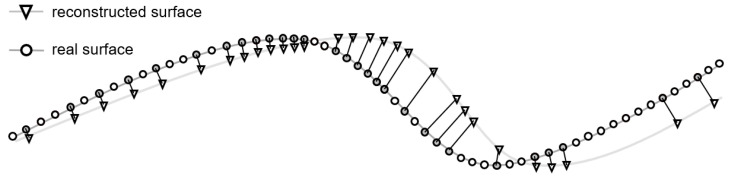
Visualization of criteria for quantitative evaluation.

The quantitative evaluation of a reconstructed surface requires the ground truth of the reference model. Most authors measure the accuracy by computing the reconstruction error, which is described as the Euclidean distance (see Figure 3: black lines) between each reconstructed surface point (see Figure 3: triangles) and its corresponding closest point on the real surface (“corresponding point pair”, see Figure 3: grey colored circles). The following metrics could be applied to evaluate the distance: mean absolute error (MAE), magnitude of relative error (MRE), standard deviation (STD), root mean squared error (RMSE), root mean squared logarithmic error (RMSE log), absolute error (AbsRel), and squared error (SqRel) (see Table 2). The MAE and the RMSE can be found in most articles, which is why we focus on these metrics [7].

Next to accuracy, there are additional criteria for the quantitative evaluation. Ref. [42] names point density, surface coverage, and robustness. The point density can be described as the total number of reconstructed points within the region of interest (see Figure 3: number of triangles). The surface coverage describes the portion of the object covered by reconstructed points (see Figure 3: ratio of triangles to circles). It is calculated as the ratio of ground truth points with a corresponding neighbor on the reconstructed surface to the total number of ground truth points. For the robustness evaluation, the performance is measured when object distance and viewing angle vary, and when smoke and blood are present. The precision is defined as the distance between the reconstructed points and the corresponding points on the fitted surface $p_{i}$.

## 3. Results

**Figure 4:**
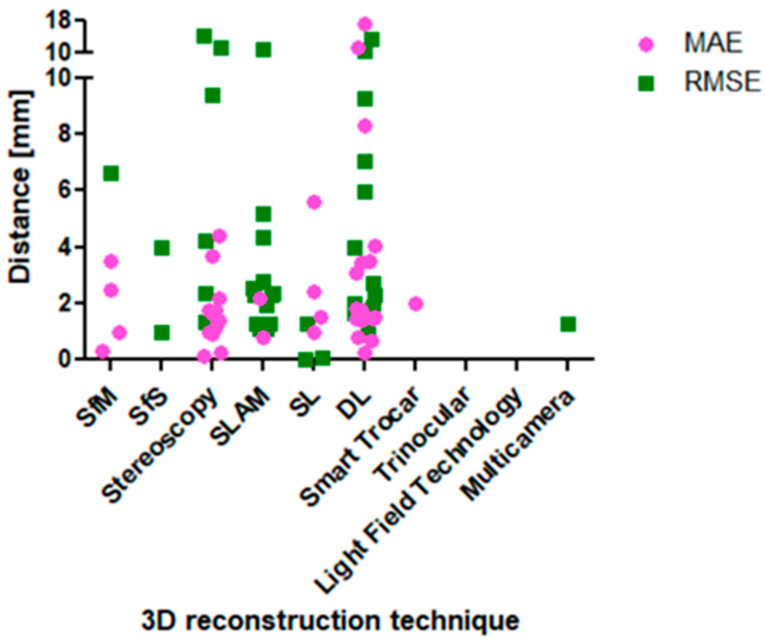
Visualization of the RMSE and the MAE values in relation to the image-based 3D reconstruction technique.

This review collects the reconstruction error of different image-based 3D reconstruction techniques to point out the technique with the highest accuracy. Table 3 lists the results, and Figure 4 visualizes these, from which three key findings can be stated. Some articles used publicly available datasets for evaluation, which are called Hamlyn centre endoscopic/laparoscopic dataset (in vivo patient datasets and validation datasets) [98], KITTI (Karlsruhe Institute of Technology and Toyota Technological Institute) (traffic scenarios) [99], and SCARED (stereo correspondence and reconstruction of endoscopic data) (datasets of fresh porcine cadaver abdominal anatomy using a da Vinci Xi endoscope and a projector) [15]. KITTI is most popular in the research of mobile robots and autonomous driving. The other two datasets are popular for endoscopic and laparoscopic research.

(1) Based on the RMSE and MAE data set, two SL, one SfM, one stereo vision, and one DL approach deliver an accuracy below one millimeter. Refs. [26,88] could deliver RMSE values below one millimeter. The SL approach in Ref. [88] is based on stereo images and achieved the lowest reconstruction error of 0.0078 mm (plate and cylinder as reference object), while Ref. [26] results in 0.07 mm RMSE (cylinder). Based on the MAE data set, a SfM (0.15 mm) (ex vivo bovine liver), a stereo vision (0.23 mm) (in vitro porcine heart images), and a DL approach (0.26 mm) (Hamlyn centre dataset) delivered the highest accuracy below one millimeter [17,55,74]. When only focusing on intraoperative organ data as reference objects and RMSE values, the best results are achieved by a SLAM approach with a value of 1.1 mm [79] (in vivo porcine abdominal cavity).

(2) Even within similar 3D reconstruction techniques, the RMSE and the MAE vary strongly. The RMSE values of the techniques stereo vision, SLAM and DL differ in a range between one millimeter and more than ten millimeters. The stereo vision approaches of Refs. [56,58,63,73] generated results between 1.31 mm and 4.21 mm, whereas Ref. [61] achieved 9.35 mm. The SLAM approaches delivered values in the range of 1.1 mm to 4.32 mm [73,76,77,79], whereas Ref. [54] results in 10.78 mm. The DL approaches generated RMSE values between 1.62 mm and 5.95 mm [16,17,19,20,22,23,73], whereas two articles present 9.27 mm and 13.18 mm [18,24].

(3) Submillimeter accuracy could only be reached by 6.5% of the articles (RMSE). As it can be seen in Figure 4 and Table 3, only two articles could achieve RMSE values below one millimeter [26,88], twelve articles could achieve RMSE values between one and two millimeters [12,13,16,17,19,22,23,63,79,81,82,86], six articles between two and three millimeters [22,23,73,77,81,82], four articles between three and five millimeters [10,20,58,76], six articles between five and ten millimeters [15,18,54,61,73,82], and four articles above ten millimeters [23,24,54,75]. Thus, most articles delivered values between one and three millimeters.

## 4. Discussion

This chapter debates the three key findings of the former chapter and three more aspects.

(1) Based on the RMSE and MAE data set, two SL, one SfM, one stereo vision, and one DL approach deliver an accuracy below one millimeter. This statement is valid if the validation scenario, which differs between the reviewed articles, is disregarded. The validation scenario must be considered to decide if it reflects the real application. The reference object is part of the validation scenario and should be considered to give a statement about realistic performance expectations. In the end, it is of little interest for an application in laparoscopy to know the performance of an algorithm on simple objects like cylinders and planes. This does not reveal much about how it performs for the actual application, which is a much more complex situation. The 3D reconstruction algorithms must also perform when there is non-rigid movement in the relevant scene, something which is indeed the case in laparoscopy. Although an algorithm may have excellent performance ex vivo for a non-moving organ, it may become very inaccurate when non-rigid movement is present in the scene. An image-based 3D reconstructions technique in laparoscopy must cope with non-rigid motion. If one technique operates on single images, it might not be badly affected by it. Whereas if one applies multiple images, as SfM for example, it may be. Any multi-view-based model assuming a rigid scene may suffer.

However, it is a necessary criterion for an algorithm to perform on simple objects. The performance test on in vivo organs must follow (sufficient criterion). For the sake of completeness, this article includes the results of all different reference objects.

**Figure 5:**
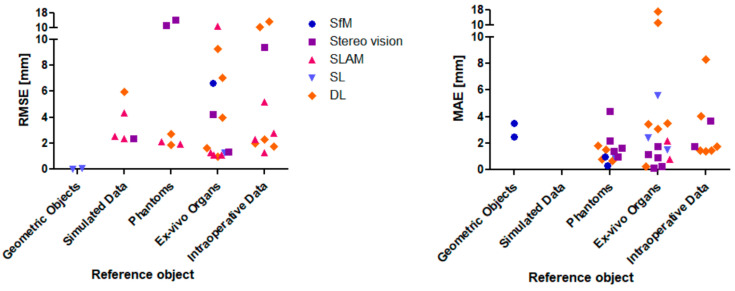
Visualization of the RMSE values in relation to the 3D reconstruction technique and the reference object (**left**); visualization of the MAE values in relation to the 3D reconstruction technique and the reference object (**right**).

Figure 5 visualizes the RMSE and MAE in dependence of the 3D reconstruction method and the reference object. (Simple) geometric objects, simulated environments, phantom organs, ex vivo organs, and intraoperative data could be found in the literature. In this order, the intraoperative data are the most complex and challenging to reconstruct. Thus, the expectation is that the reconstruction error increases with increasing complexity of the reference object. Due to a high variation of the reconstruction error, this tendency can only be observed slightly in Figure 5. The two SL approaches achieving RMSE values of 0.07 mm [26] and 0.0078 mm [88] can be found in the category of geometric objects of the left diagram of Figure 5 (plate and cylinder as reference object, left diagram in Figure 5). It would be supportive to validate these results on in vivo organs. In contrast to that, the SL approach of Ref. [86] results in 1.28 mm (RMSE, category ex vivo organ in the left diagram of Figure 5). The three mentioned articles did not measure the MAE, but Ref. [87] did. There, a monocular SL system was built and reached 1.0 ± 0.4 mm MAE (phantom as reference object, right diagram in Figure 5), which is close to submillimeter accuracy. Here, it would be supportive to measure the RMSE as well because it weighs outliers more and is the more critical and representative metric. In summary, the performance of SL approaches cannot be stated when only tested on simple geometric objects, phantoms, or ex vivo organs—additionally intraoperative data are necessary.

Ref. [55] applied a SfM approach and Ref. [74] applied a stereo vision approach—both only measuring the MAE (0.15 mm and 0.23 mm), but not the RMSE. Both did not evaluate their algorithms on intraoperative data, but on ex vivo organs. To confirm the high performance, it requires in vivo data and RMSE computation.

**Figure 6:**
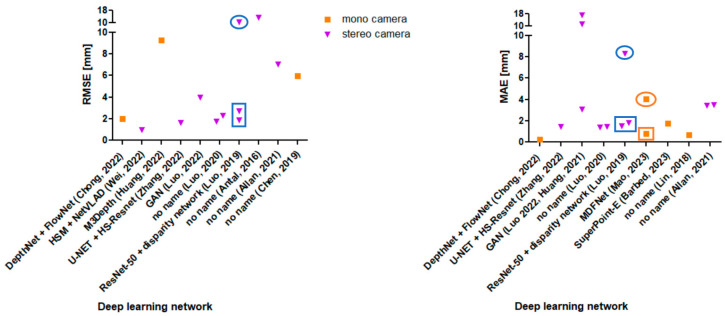
Visualization of the RMSE values from the Refs. [15,16,17,18,19,20,22,23,24,73] in relation to the deep learning network and the number of cameras (**left**); Visualization of the MAE values from the Refs. [14,15,17,19,20,21,22,23,92,93] in relation to the deep learning network and the number of cameras (**right**); the rectangle boxes marks the data from phantom organs and the circles mark the data from in vivo images.

Ref. [17] applied a DL approach and measured 1.98 mm RMSE, which is the highest accuracy compared to the other DL approaches (see left diagram in Figure 5). Moreover, it is validated on intraoperative data, which declare this approach as promising. The authors used the networks DepthNet and FlowNet with monocular images as input (see Figure 6).

Finally—based on the former presented dataset—the SLAM approach by [78] can be stated as the most promising approach because the RMSE value is close to submillimeter accuracy (1.1 mm) and the authors used intraoperative laparoscopic data. However, more data are needed for comparison purposes to either confirm or refute the good performance of the SL and DL approaches.

(2) Even within similar 3D reconstruction techniques, the RMSE and the MAE values vary strongly. In some articles, stereo vision, SLAM, and DL approaches achieve higher accuracies than in other articles. The results show a high variation. The influence of the reference object can be seen by a deep dive into the DL approaches (see Figure 6 left and right). Refs. [23,92] validated their algorithms by using both phantom organs (see data point in rectangles in Figure 6 left and right) and intraoperative images (see ovals in Figure 6 left and right). There, the reconstruction results of the intraoperative images are worse than those of the phantom organs (by factor ~5). If there was a factor to estimate the accuracy for intraoperative data based on simple objects, this would be supportive for future researchers, as they could start their algorithm evaluation on simple objects. If the performance already fails then, they can save the effort of further in vivo evaluations.

Figure 6 shows a diagram in which DL approaches are separated into either stereoscopic or monocular input images. The expectation was that stereoscopic input images deliver higher accuracies due to the second camera perspective. This statement cannot be confirmed, as there are too few data. Moreover, the performance of the different DL networks might have an influence on the results as well.

(3) Submillimeter accuracy could only be reached by 6.5% of the articles (RMSE).

In a laparoscopic intervention, movement (heartbeat, breathing, intestinal peristalsis), organ specularity, and occlusions (instruments, blood, smoke) challenge the 3D reconstruction algorithms to reach submillimeter accuracy [7]. It can be achieved when using geometric objects as a reference [26,88]. Probably, some applications do not require submillimeter accuracy. The supervision of preoperative 3D models with intraoperative laparoscopic images for image-guided navigation for example needs less accuracy if the laparoscope is guided manually [7]. Ref. [100] reports that a maximum error of 1.5–2 mm is acceptable. Autonomous robot-assisted interventions require a higher accuracy. Ref. [101] reports that the doctor’s hand can achieve an accuracy of 0.1 mm, which should also be met by the robot.

(4) Accuracy might depend on several influence factors. Due to the inability to explain the high variation of the reconstruction error, there are assumptions that the different validation scenarios might be responsible for it. The following subjects are listed as possible influence factors:

- Reference object (geometric objects vs. simulated data vs. ex/in vivo data).
- Method of ground truth acquisition (CT data vs. laser scanner vs. manual labelling).
- Method of camera localization (known from external sensor vs. image-based estimation).
- Number of frames (single shot vs. multiple frames/multi view).
- Image resolution (the higher the more 3D points).
- Number of training data (relevant for DL approaches.
- Implemented algorithms (e.g., SIFT vs. SURF, ORB-SLAM vs. VISLAM).

To prove the above-mentioned influence factors, more data are required as well as a comparison of different image-based 3D reconstruction techniques under the same validation scenarios.

(5) Additional aspects for validation. Not only the quantitative metrics should be considered for validation. When choosing a 3D reconstruction method, the computation time, the necessity of additional hardware, the investment costs, and the compatibility with the standard equipment in clinics should be evaluated [7]. When looking at the computation time, a few articles—DL and stereo vision—claim to achieve real time with around 20–30 fps or close to real time with 11–17 fps [16,19,59,60,74]. The aspect of additional hardware compatibility with the standard equipment of clinics and the investment costs correlate. Stereo vision requires a second camera, which, compared to monocular laparoscopes, is more expensive, and thus, not every clinic provides these. SL requires additional hardware as well (projecting light source) and standard laparoscopes do not contain SL. Regarding this aspect, SfM, SfS, SLAM, and DL (monocular images as input) would be the better choice.

(6) Recommendations. In future research, it would be recommendable to follow a standard evaluation procedure. First, the evaluation should be made either with a MIS data set (e.g., data set of Hamlyn centre, SCARED), which includes ground truth, or with other intraoperative image data. Second, in case of an own image acquisition, including ground truth, these data should be published so that other researchers have access. Third, the MAE and the RMSE of the reconstruction error must be measured. Fourth, the ground truth must be as close as possible to the real shape, which is best by either CAD data, CT or MRT data, or reliable 3D scanners. Fifth, the influence factors should be documented. Refs. [54,57,73,85] included a comparison of different approaches, which are good examples for future research articles.

## 5. Conclusions

This review (2015–2023) presented laparoscopic image-based 3D reconstruction techniques and compared their accuracy to name the most promising one, from which SLAM, DL, and SL achieved the lowest reconstruction error (RMSE) in submillimeter and near submillimeter range. The SLAM approach reached 1.1 mm RMSE (in vivo porcine abdominal cavity), the DL approach reached 1.98 mm RMSE (Hamlyn centre dataset), and the SL approach reached 0.0078 mm RMSE (plate and cylinder). For the evaluation of the reconstruction techniques, the reference object must be rated if it is representative for laparoscopy—the actual application. Any viable approach should have a low reconstruction error for simple objects. However, simple models or even more complex ones—if they are at rest or rigid—as basis for comparison can be deceptive. Even if one approach has on-par or even better performance than another, the former can perform significantly worse in a realistic situation if it cannot cope with non-rigid motion, whereas the latter is not affected. Following this, a plate, a cylinder, and even ex vivo organs are not sufficient for the evaluation. Thus, it requires intraoperative data to confirm an approach’s performance.

Finally, it is recommendable for future researchers to always consider both the MAE and the RMSE of the reconstruction error, to apply their algorithms in vivo organs, publicly available MIS datasets, or publish own datasets, and to compare the own results with other approaches.

## Figures and Tables

**Table 1:** Overview of 3D reconstruction techniques in laparoscopy since 2015.

Image-Based 3D Reconstruction Technique	2015	2016	2017	2018	2019	2020	2021	2022	2023	Total
SfM [9,47,48,49,50,51,52,53,54,55]	1	1	5	1	1	1				10
SfS [10]	1									1
Stereo vision [4,56,57,58,59,60,61,62,63,64,65,66,67,68,69,70,71,72,73,74,75]	4	2	3	6		3		1	1	20
SLAM [13,54,73,76,77,78,79,80,81,82]			2	2	1	2	1		2	10
SL [5,26,83,84,85,86,87,88,89,90,91]	2	1	2	3	1	1		1		11
DL [14,15,16,17,18,19,20,21,22,23,24,35,36,37,38,39,92,93,94,95]		1		1	2	1	5	6	4	20
Smart Trocar [27]			1							1
Trinocular [11]			1							1
Light Field Technology [28]			1							1
Multicamera (10x) [12]					1					1
ToF										0
Total	8	5	15	13	6	8	6	8	7	76

**Table 2:** Overview of metrics for quantitative evaluation with reconstructed point ${\hat{y}}_{i}$, corresponding closest point on the real surface $y_{i}$, number of scanned points N, and mean value μ [16].

Metrics	Definition	Target Value
MAE	$\frac{1}{N}\sum_{i=1}^{N}\left|{\hat{y}}_{i}-y_{i}\right|$	0.0 mm
MRE	$\frac{1}{N}\sum_{i=1}^{N}\frac{\left|{\hat{y}}_{i}-y_{i}\right|}{y_{i}}$	0.0 mm
STD	$\sqrt{\frac{1}{N}\sum_{i=1}^{N}{\left({\hat{y}}_{i}-\mu \right)}^{2}}$	0.0 mm
RMSE	$\sqrt{\frac{1}{N}\sum_{i=1}^{N}{\left({\hat{y}}_{i}-y_{i}\right)}^{2}}$	0.0 mm
RMSE log	$\sqrt{\frac{1}{N}\sum_{i=1}^{N}{\left(\log{\hat{y}}_{i}-\log y_{i}\right)}^{2}}$	0.0 mm
SqRel	$\frac{1}{N}\sum_{i=1}^{N}\frac{{\left|{\hat{y}}_{i}-y_{i}\right|}^{2}}{{\hat{y}}_{i}}$	0.0 mm

**Table 3:** Collection of the reconstruction error of the 48 reviewed articles, including the 3D reconstruction technique, the reference object, the ground truth, the mean absolute error (MAE), the root mean squared error (RMSE), and the author. Data marked with * are delivered via mail by the authors.

3D Reconstruction Technique	Reference Object	Ground Truth	MAE ± STD [mm]	RMSE ± STD [mm]	Author
Structure-from-Motion (SfM)	15 seq. of 3 cirrhotic liver phantoms	Optical tracking system	0.3–1.0	-	[9]
	Ex vivo bovine liver	PhotoScan (SL projecting system + mechanical arm for laparoscope guidance)	0.15 ± 0.05	-	[55]
	(1) Paper (2) T-shirt (3) Stereo laparoscopic video	RGB-D sensor	2.5–3.5	-	[48]
	Dataset of moving camera of static surgical scene w/known camera pose	Space Spider (white light 3D scanner)	-	6.628	[54]
Shape-from-Shading (SfS)	Laparoscopic (mono) surgery video data	Two consecutive frames after ICP	-	1.0–4.0	[10]
Stereo vision	Liver phantom model	Intraoperative CT data	4.4 ± 0.8	-	[4]
	Publicly available phantom dataset	CT data	-	5.45 Pixel	[56]
	Ex vivo porcine liver and Hamlyn centre dataset	Included in dataset	1.14; 1.77–3.7	-	[57]
	Ex vivo porcine liver	CT data	-	4.21 ± 0.63	[58]
	Hamlyn centre dataset	Included in dataset (made by Library for efficient large-scale Stereo Matching (LIBELAS))	1.75	-	[59]
	Phantom model	Eight points on phantom for distance measurement	1.0	-	[60]
	In vivo liver with and without pneumoperitoneum, but with a tube disconnected to stop breathing)	CT data (intraoperative)	-	9.35 ± 2.94	[61]
	Phantom surgical cavity	Space Spider 3D Scanner		11.2547; 13.9759	[75]
	Phantom model	3D scanner	1.4 ± 1.07	-	[62]
	Ex vivo tissue	External tracking system	0.89 ± 0.7	1.31 ± 0.98	[63]
	Phantom heart	CT data	2.16 ± 0.65	-	[68]
	In vitro porcine heart images	CT data	0.23 ± 0.33	-	[74]
	Liver phantom	CT data	1.65 ± 1.41	-	[69]
	Simulated MIS scene	Known scene dimensions	-	2.37	[73]
Simultaneous Localization and Mapping (SLAM)	Dataset of moving camera of static surgical scene w/unknown camera pose	Space Spider (white light 3D scanner)	-	10.78	[54]
	Ex vivo porcine livers	Electromagnetic trackers	0.8–2.2 ± 0.4–0.7	1.1–1.3 * ± 0.6–0.7 *	[13]
	Simulated MIS scene	Known scene dimensions	-	4.32	[76]
	Porcine in vivo data	CT data	-	2.8	[77]
	Synthetic abdominal cavity box (silicone)	Known scene dimensions	-	1.94; 2.13;	[81]
	Hamlyn centre dataset	Included in dataset	-	1.3; 2.3; 5.2	[82]
	Simulated MIS scene	Known scene dimensions	-	2.54	[73]
	In vivo porcine abdominal cavity	CT data	-	1.1	[79]
Structured Light (SL)	Cylinder surface w/diameter of 22.5 cm	Known object dimensions	-	0.07	[26]
	Porcine cadaver kidney	Measurement of cut in kidney	2.44 ± 0.34	-	[84]
	Ex vivo kidney	Certus optical tracker stylus	5.6 ± 4.9 1.5 ± 0.6	-	[85]
	Ex vivo porcine liver and kidney	CT data	-	1.28	[86]
	Patient-specific phantoms built by rapid prototyping	Known object dimensions	1.0 ± 0.4	-	[87]
	(1) Plate (2) Cylinder	Known object dimensions	-	0.0078	[88]
Deep Learning (DL)	KITTI dataset	Included in dataset	-	5.953	[73]
	Silicone heart phantom	MCAx25 handheld scanner	0.68 ± 0.13	-	[14]
	SCARED dataset test set 1, test set 2	Included in dataset (SL + da Vinci Xi kinematics)	3.44; 3.47	7.01 * (Rosenthal)	[15]
	SCARED dataset test set 1, test set 2	Included in dataset	-	1.0	[16]
	Hamlyn centre dataset and additional monocular laparoscopic image sequences	Included in dataset and CT data	0.26	1.98	[17]
	SCARED dataset test set 1, test set 2	Included in dataset	-	9.27	[18]
	Hamlyn centre dataset	Included in dataset	1.45 ± 0.4	1.62 ± 0.42	[19]
	SCARED dataset test set 1, test set 2	Included in dataset	3.05	3.961 * ± 1.237 *	[20]
	SCARED dataset test set 1, test set 2	Included in dataset	11.23; 17.42	-	[21]
	Real colonoscopy videos	Points being reprojected onto images	1.75 ± 0.07	-	[93]
	In vivo porcine hearts	Pre-operative CT data	1.41 ± 0.42 1.43 ± 0.47	1.77 ± 0.51 2.27 ± 0.39	[22]
	Hamlyn centre dataset and three points on heart phantom model	Artec Eva scanner	4.047 0.78 ± 0.22	-	[92]
	SCARED dataset test set 1, test set 2	Included in dataset	2.64 ± 1.64 Pixel	5.47 ± 1.46 Pixel	[94]
	Heart phantom model and Da Vinci dataset	Rigid confidence measurement	1.49 ± 0.41 1.84 ± 0.4 8.3 ± 3.1	1.9 ± 0.38 2.69 ± 0.58 10.5 ± 3.7	[23]
	Laparoscopic cardiac dataset	Included in dataset	-	13.18	[24]
Smart Trocar^®^	3D-printed plastic sphere model. Surface is divided into 1 cm^2^ squares	Known object dimensions	2.0	-	[27]
Trinocular	Cartilage of pig knee joint	Vialux zSnapper (3D fringe projection system)	±1.1	-	[11]
Multicamera (10 cameras)	Phantom surgical scene	Space Spider	-	1.29	[12]

## Data Availability

The data that supports the findings of this work are available from the corresponding author.
